# Analyzing the Korean reference genome with meta-imputation increased the imputation accuracy and spectrum of rare variants in the Korean population

**DOI:** 10.3389/fgene.2022.1008646

**Published:** 2022-11-24

**Authors:** Mi Yeong Hwang, Nak-Hyeon Choi, Hong Hee Won, Bong-Jo Kim, Young Jin Kim

**Affiliations:** ^1^ Division of Genome Science, Department of Precision Medicine, National Institute of Health, Cheongju-si, South Korea; ^2^ Department of Digital Health, Samsung Advanced Institute for Health Sciences and Technology (SAIHST), Samsung Medical Center, Sungkyunkwan University, Seoul, South Korea

**Keywords:** whole-genome sequencing (WGS), variant, genotype imputation, meta-imputation, Korean reference genome

## Abstract

Genotype imputation is essential for enhancing the power of association-mapping and discovering rare and indels that are missed by most genotyping arrays. Imputation analysis can be more accurate with a population-specific reference panel or a multi-ethnic reference panel with numerous samples. The National Institute of Health, Republic of Korea, initiated the Korean Reference Genome (KRG) project to identify variants in whole-genome sequences of ∼20,000 Korean participants. In the pilot phase, we analyzed the data from 1,490 participants. The genetic characteristics and imputation performance of the KRG were compared with those of the 1,000 Genomes Project Phase 3, GenomeAsia 100K Project, ChinaMAP, NARD, and TOPMed reference panels. For comparison analysis, genotype panels were artificially generated using whole-genome sequencing data from combinations of four different ancestries (Korean, Japanese, Chinese, and European) and two population-specific optimized microarrays (Korea Biobank Array and UK Biobank Array). The KRG reference panel performed best for the Korean population (*R* ^2^ = 0.78–0.84, percentage of well-imputed is 91.9% for allele frequency >5%), although the other reference panels comprised a larger number of samples with genetically different background. By comparing multiple reference panels and multi-ethnic genotype panels, optimal imputation was obtained using reference panels from genetically related populations and a population-optimized microarray. Indeed, the reference panels of KRG and TOPMed showed the best performance when applied to the genotype panels of KBA (*R* ^2^ = 0.84) and UKB (*R* ^2^ = 0.87), respectively. Using a meta-imputation approach to merge imputation results from different reference panels increased the imputation accuracy for rare variants (∼7%) and provided additional well-imputed variants (∼20%) with comparable imputation accuracy to that of the KRG. Our results demonstrate the importance of using a population-specific reference panel and meta-imputation to assess a substantial number of accurately imputed rare variants.

## 1 Introduction

Population-scale sequencing of reference datasets has led to an increased number of variants from multi-ethnic populations (Auton et al., 2015; Karczewski et al., 2020; Taliun et al., 2021). The discovered variants from the reference datasets provide valuable information for studying potential drug targets, genetic diversity across populations, population-specific rare variants with strong genetic effects, and hidden associated variants responsible for missing heritability. In addition, these data can be used as a reference panel for genotype imputation (essential for genome-wide association analysis) by enabling the estimation of untyped markers in single-nucleotide polymorphism (SNP) microarrays (Das et al., 2016). Previous studies have had a Euro-centric bias, which may result in poor disease prediction for non-European populations; therefore, more genomic data are required from such populations (Martin et al., 2019).

To date, the largest proportion of whole-genome sequencing datasets has been derived from populations with European ancestries, including datasets in the Haplotype Reference Consortium (HRC, *n* = 32,470) (McCarthy et al., 2016), the Trans-Omics for Precision Medicine (TOPMed, *n* = 97,256) program (Taliun et al., 2021), the Genome Aggregation Database (gnomAD, *n* = 141,456) (Karczewski et al., 2020), and the United Kingdom Biobank (UKB) (*n* = 200,000) (Halldorsson et al., 2021). The 1000 Genomes Project Phase 3 (1KGP3) reference panel (*n* = 2,504) is the most widely used multi-ethnic reference dataset, although it has a relatively small number of samples per population (Auton et al., 2015). Numerous reports have described the use of reference datasets dedicated to East Asian populations, including the China Metabolic Analytics Project (ChinaMAP, *n* = 10,155) (Cao et al., 2020; Li et al., 2021), the GenomeAsia 100K Project (GAsP, *n* = 1,654) (GenomeAsia, 2019), the Northeast Asian Reference Database (NARD, *n* = 1,779) (Yoo et al., 2019), the Korean Genome Project (KGP, *n* = 1,094) (Jeon et al., 2020), and the Japanese whole-genome sequence (JWGS, *n* = 2,234) dataset (Okada et al., 2018). However, substantially fewer whole-genome sequences are available for East Asians than for Europeans, and more datasets are warranted to explore less frequent variants in East Asians. Despite previous efforts, the ability to identify diseases associated with novel variants using sequencing datasets remains limited, owing to the relatively small number of samples analyzed in most sequencing projects, other than the TOPMed and UKB projects (Halldorsson et al., 2021; Taliun et al., 2021).

Genotype imputation using the aforementioned reference datasets as reference panels enabled us to comprehensively interrogate rare variants (minor-allele frequency [MAF] <1%) and insertions/deletions (INDELs), which are difficult to detect using most genotyping arrays (Das et al., 2016; Li et al., 2021; Taliun et al., 2021). The imputation analysis was used to estimate untyped markers in the SNP microarray based on adequate haplotypes from the reference panels and to expand the number of variants in the SNP microarray dataset up to those of the reference panel (Auton et al., 2015). The approach enhanced the association-mapping power of existing genotype data with no additional genotyping cost. Imputation analysis can be more accurate with a population-specific reference panel (Kim et al., 2015; Yoo et al., 2019; Jeon et al., 2020; Li et al., 2021) or a multi-ethnic reference panel with a large number of samples, such as TOPMed (Taliun et al., 2021). Indeed, analysis of the ChinaMAP dataset revealed the importance of using a population-specific reference panel by showing an increased imputation accuracy over that of the TOPMed reference panel, which has approximately 10-fold more samples with multi-ethnic ancestries (Li et al., 2021). Therefore, a large-scale population-specific reference panel is warranted to provide high imputation accuracy for a specific population.

The National Institute of Health, Republic of Korea, has initiated the Korean Reference Genome (KRG) project to facilitate Korean genome research and identify a spectrum of variants in the Korean population, a major ethnic group of East Asia. With the KRG project, approximately 20,000 whole-genome sequences will be analyzed to provide a comprehensive catalog of putative disease-causing variants as well as a reference panel useful for expanding Korean genome research to include rarer variants with enhanced imputation accuracy.

Through the KRG pilot project, we aimed to comprehensively assess genetic characteristics of the Korean population, the discovery of novel genetic variants, and the effect of an imputation reference panel for an association study. Here, we describe the catalog of variants generated during the pilot phase of the KRG project (*n* = 1,490) and its imputation performance compared to those of pre-existing reference panels, such as TOPMed, ChinaMAP, NARD, and GAsP. For assessing imputation performance in multi-ethnic populations comprising different combinations of reference panels and genotype panels, mimicked genotyped panels artificially created from the original sequencing data, were derived using sequenced samples from Chinese, Japanese, and European individuals (the 1KGP3 reference panel). In addition, we studied the possibility of increasing the imputation accuracy via a meta-imputation approach, a comprehensive approach to statistically merging imputation results from multiple different reference panels, which showed a comprehensive increase in the imputation performance without the need for assessing individual-level genotype data in the reference panels (Yu et al., 2022).

## Materials and methods

### Study population

For the pilot phase of the KRG project, 1,499 samples were subjected to whole-genome sequencing. These samples were obtained from 400 healthy volunteers (recruited for the KRG project) and 1,099 participants (selected from among the Korean Genome and Epidemiology Study [KoGES] cohort). All participants provided written informed consent. Genomic DNA samples from the participants were subjected to whole-genome sequencing using the Illumina HiSeq 2000 and HiSeq 2500 platforms for the KRG and KoGES samples, respectively. Approval to analyze the KRG and KoGES samples was granted by the institutional review board of the Korea Disease Control Prevention and Control Agency (approval number 2022-02-04-P-A).

### Construction of the KRG reference panel

A schematic representation of the construction of the KRG reference panel is provided in Supplementary Figure S1. Low-quality bases were trimmed using the Trimmomatic software (version 0.39) (Bolger et al., 2014). Quality-controlled sequence reads were aligned after removing polymerase chain reaction-based duplicates, and variants were called using DRAGEN™ (Miller et al., 2015). The mapping depths ranged from 8.1× to 50.8× (mean depth: 29.0×). Raw variants were further processed using the Variant Quality Score Recalibration (VQSR) algorithm of the Genome Analysis Toolkit (version 4.2.1.0) (McKenna et al., 2010). Based on the results of our VQSR analysis, variants were included if they were marked as “PASS”, “VQSRTrancheSNP99.90to100.00”, or “VQSRTrancheINDEL99.90to100.00”. Among the 1,499 samples, 1,499 independent samples were retained after excluding second-degree relatives based on the results from KING v2 (Manichaikul et al., 2010). All samples met the missing rate threshold <5%. Possible low-quality variants among the 1,490 samples were removed by filtering out variants meeting one of the following criteria: a Hardy-Weinberg equilibrium *p*-value of <10^−6^, a missing rate of >5%, and location within low-complexity regions as determined from a repeat-masker table (UCSC Genome Browser).

To construct the KRG reference panel, additional quality-control analysis was applied to the dataset to prevent the possibility of discovering false variants found in sequences with a relatively low depth (∼10×). Variants were further removed if they contained indels, >10 multiple alternative alleles, or singletons (allele count = 1). Then, variants were retained in the reference panel in cases where the variant was included in more than two out of five public whole-genome sequencing datasets (1KGP3, HRC, TOPMed, GAsP, and ChinaMAP). Consequently, 13,637,761 variants from 1,490 samples were retained for further analysis. The quality-controlled dataset was phased using the SHAPEIT4 (version 4.2.2) (Delaneau et al., 2019), and a phased variant call format file was used as the reference panel for subsequent imputation analysis using Minimac4 (version 1.0.2) (Das et al., 2016).

### Population genetic analyses

Population genetic analyses was performed using a subset of 5.4 K variants among commonly available variants from KRG and 1KGP3. These autosomal variants were separated by 0.5 M among commonly available variants across populations to avoid selection bias by using linkage disequilibrium (LD) based marker selection in multi-ethnic populations. Principal component analysis (PCA) was conducted using flashPCA2 (Abraham et al., 2017). Population structure was inferred by using ADMIXTURE (Alexander et al., 2009). Using the —cv option of ADMIXTURE, the subpopulation value K parameter was selected as minimizing cross-validation error rate. For PCA and ADMIXTURE analysis, 5,388 variants were used as an input and the results were plotted against subpopulation information of individuals of KRG and 1KGP3. The inter-population variation in LD was analyzed by using varLD (Ong and Teo, 2010). For varLD analysis, commonly available 5,635,734 variants in both KRG and Europeans from 1KGP3 were used. Using raw scores from varLD analysis, we calculated standardized varLD scores using the R script provided in the varLD website (https://blog.nus.edu.sg/sshsphphg/varld/). The regions of top 1% variation in LD were selected if the region had the varLD score more than the value of top 1% percentile among standardized varLD scores.

#### Genotype panel

For comparison analysis of imputation performance, eight different genotype panels were constructed based on four multi-ethnic populations by mimicking two population-optimized biobank arrays, including the Korea Biobank Array (KBA) and the UKB array (Supplementary Table S1). The UKB array was designed to contain markers for providing genome-wide coverage in Caucasian European populations (UKB resource #146640). The four populations comprised Korean (KOR, *n* = 208), Japanese in Tokyo (JPT, *n* = 104), Chinese (CH, *n* = 208), and European (EUR, n = 208) panels. The KOR panel was created by extracting variants from 208 whole-genome sequences from Korean subjects who were not included in the construction of the reference panel. The JPT, CH, and EUR panels were constructed by retrieving variants from 1KGP3. For each genotype panel, the KBA and UKB arrays were artificially created by extracting variants from the whole-genome sequencing data.

#### Genotype imputation

Pre-phasing imputation analysis was performed as follows. First, variants with an MAF of <1%, regarded as genotyped on the microarray, were removed from eight different genotype panels for preventing possible errors during phasing analysis. Then, the Eagle software (version 2.3) (Loh et al., 2016) was used to phase the genotype panels. TOPMed, ChinaMAP, GAsP, NARD, and KRG sequencing data were used as reference panels. For TOPMed, GAsP, and NARD, pre-phased genotype panels were uploaded to the TOPMed, Michigan, and NARD imputation servers, respectively. For ChinaMAP, phasing and imputation were performed using the default option of the ChinaMAP imputation server. For KRG, imputation was performed on pre-phased genotype panels by applying identical options used by the Michigan imputation server. Meta-imputation was performed using MetaMinimac2 (Yu et al., 2022) with various combinations of data from the TOPMed, GAsP, 1KGP3 (for KOR only) and KRG datasets but not the ChinaMAP and NARD dataset. At the time of the analysis, ChinaMAP and NARD did not support the imputation output applying meta option of Minimac4, which is a required input for meta-imputation.

### Statistical analysis

To measure the imputation performance, aggregated *R* ^2^ and the proportion of well-imputed variants were determined using Eq. 1 and Eq. 2, respectively:

$$Aggregate\;R^{2}=cor\;{\left(imputation\;allele\;dosages,\;true\;genotype\;dosages\right)\;}^{2}$$ (1)

True genotype dosages were retrieved from whole-genome sequencing datasets with identical samples.

$$\%\;of\;well-imputed\;variants=\frac{Number\;of\;imputed\;variants\;\left(estimated\;r^{2}\geq 0.8\right)}{Number\;of\;total\;imputed\;variants}\times 100$$ (2)

Plots were generated using the ggplot2 package in R (version 4.1.3).

## Results

### Variants discovered with the KRG reference panel

**FIGURE 1:**
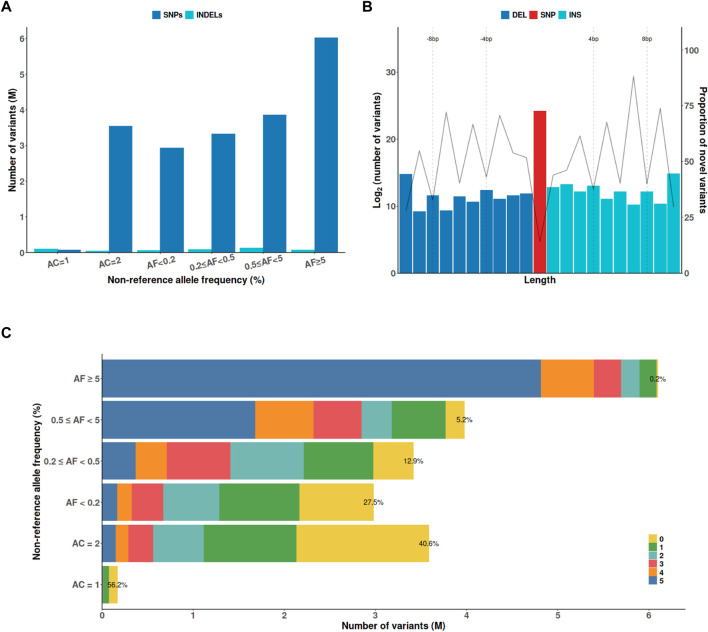
The characteristics of genetic variants discovered by the KRG project. **(A)** The *x*-axis indicates the non-reference AF. **(B)** Variants were defined as novel if they were not present in the other whole-genome sequencing datasets (1KGP3, HRC, TOPMed, GAsP, and ChinaMAP). **(C)** The numbers indicate the number of ther other whole-genome sequencing datasets that contained a variant with the indicated AF; ‘0’ indicates that a variant was only found in the KRG panel. Abbreviations: AC, allele count, AF, allele frequency; DEL, deletion; INDEL, insertion/deletion; INS, insertion; SNP, single-nucleotide polymorphism; KRG, Korean Reference Genome.

The KRG panel contained 20,220,176 variants, including 19,736,544 SNPs and 483,632 indels after initial quality-control analysis (Figure 1A). These variants comprised 115,210 protein-altering variants, including 107,916 nonsynonymous mutations, 2,370 stop gained/lost, 348 start lost, 3,326 splicing acceptor/donor mutations, 402 frameshift, and 848 in-frame indels. To analyze novel variants in the KRG panel, the variants were compared with those in five different whole-genome sequencing datasets, namely, 1KGP3, HRC, TOPMed, GAsP, and ChinaMAP (Figures 1B,C). The proportion of novel variants exclusively found in the KRG increased as the allele length increased for indels (Figure 1B), or the allele frequency (AF) decreased (Figure 1C). The KRG included a total of 166,939 (0.83%) novel functional variants that were not present in the other sequencing datasets. The KRG panel was processed by applying stringent additional quality-control analysis when constructing the reference panel for imputation analysis (See Materials and Methods). Consequently, 13,637,761 variants from 1,490 samples were retained for further analysis (Table 1).

**TABLE 1:** Spectrum of variants in the Korean Reference Genome reference panel (pilot phase).

Non-reference allele frequency	Number of variants (%)	Number of functional variants^1^ (%)
≥5%	5,872,163 (43.1)	19,924 (0.34)
0.5–5%	3,166,820 (23.2)	17,841 (0.56)
0.2–0.5%	2,206,624 (16.2)	16,095 (0.73)
<0.2%	2,392,154 (17.5)	19,595 (0.82)
Total	13,637,761	73,455 (0.54)

^a^ Functional variants: nonsynonymous mutations, gained/lost stop codons, lost start codons, splicing acceptor/donor mutations, frameshift mutations, in-frame insertions/deletions.

### Population structure of the KRG

**FIGURE 2:**
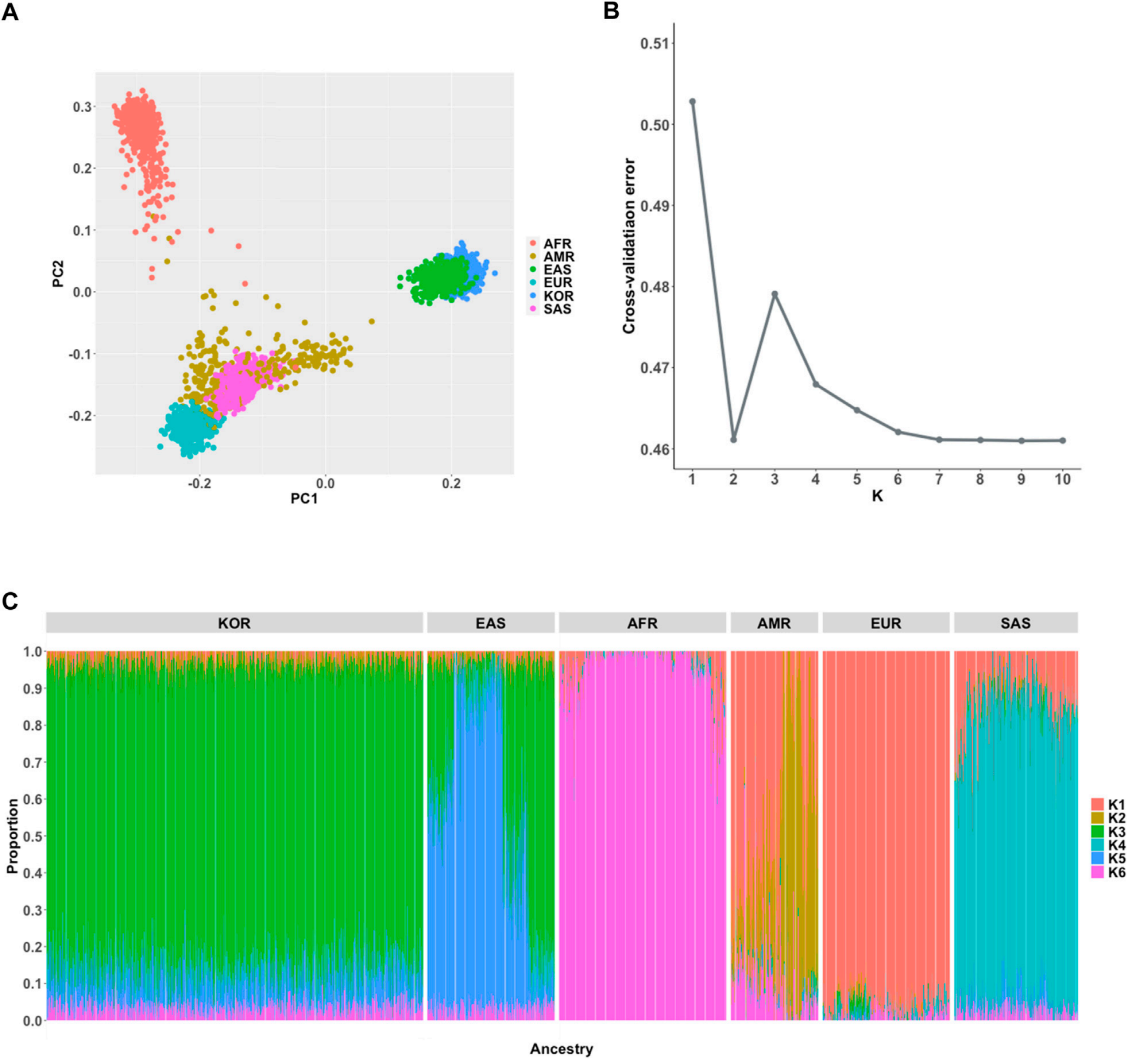
Population genetic analyses. **(A)** Principal component analysis of 6 ancestries **(B)** Estimation of an optimal number of subpopulations (K) **(C)** Distribution of the 6 ancestry components in the Korean and 1KGP3 populations (K = 6).

For comprehensive understanding of population structure of the Korean samples in the KRG compared to multi-ethnic samples from the 1KGP3 dataset, we performed population genetic analyses including PCA and population structure using ADMIXTURE software (Alexander et al., 2009). As expected, PCA analysis results showed that Korean samples were closely clustered with geographically close neighbors (Japanese and Chinese samples of East Asians (EAS) from 1KGP3) (Figure 2A). We further analyzed an individual’s proportion of ancestries using ADMIXTURE. Prior to the analyses, the optimal number of subpopulations (K) was selected as six based on cross-validation error analysis (Figure 2B). Although EAS samples were closely located and genetically clustered in the PCA result, the composition of ancestries was different among Koreans and East Asians from 1KGP3 yet shared similar ancestral components (Figure 2C). In addition, PCA analysis on only samples from EAS and KRG showed that Koreans were clearly distinguished among the East Asian samples (Supplementary Figure S2). When we applied ADMIXTURE analysis on only Korean samples for analyzing possible sub-population patterns, number of subpopulations was optimal at K = 1 for Korean samples and PCA analysis on Korean samples further supported the finding (Supplementary Figure S2). It implies that Koreans would be genetically homogeneous compared to other subpopulations in East Asians. These results were in consistent with the previously described population structure of the Korean samples (Kim and Jin, 2013; Yoo et al., 2019).

### Imputation performance of the KRG panel

**FIGURE 3:**
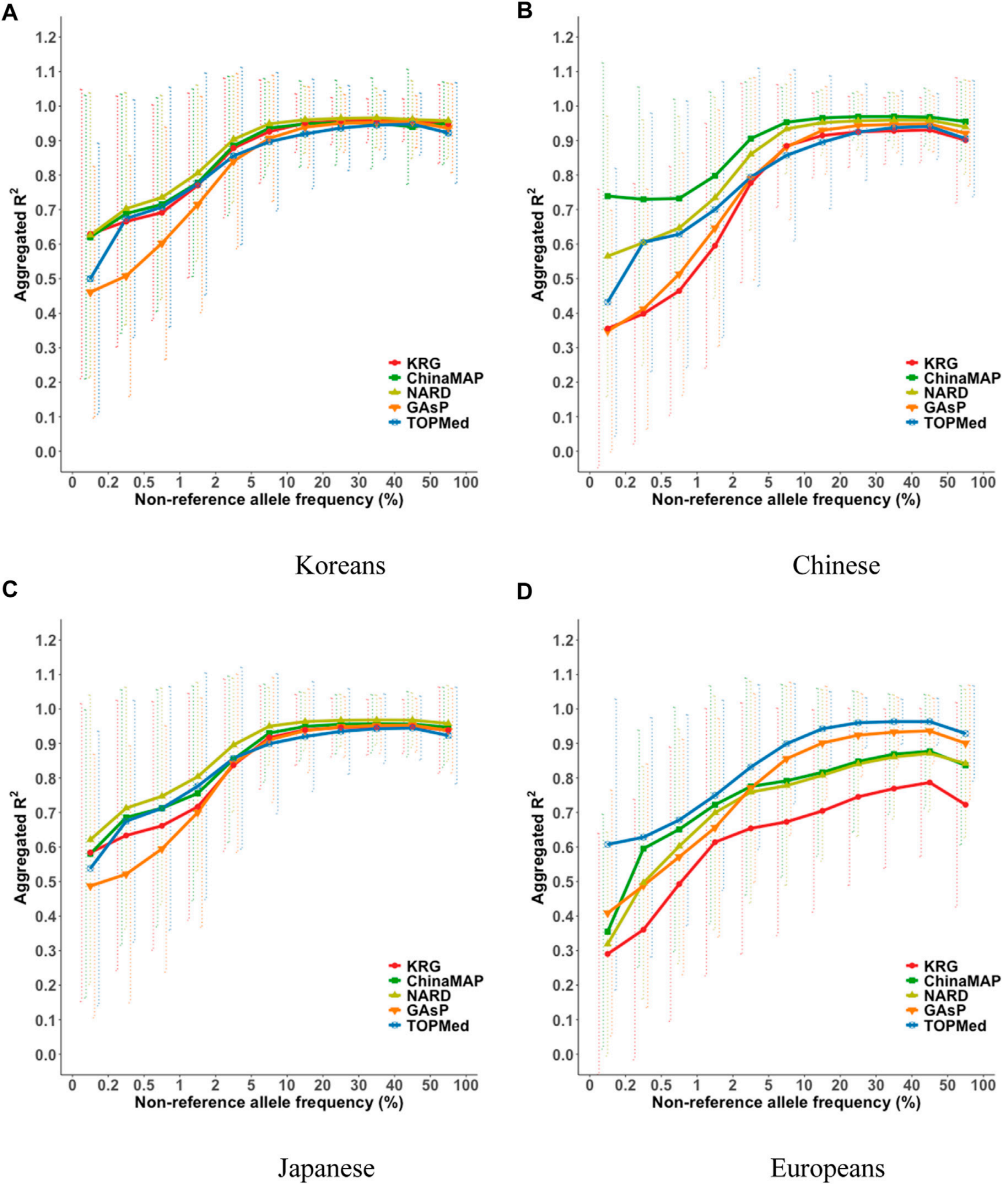
Aggregated R^2^ based on combining reference and genotype panels mimicking the KBA. The aggregated *R* ^2^ values were computed for the target sample of **(A)** Korean, **(B)** Chinese, **(C)** Japanese, and **(D)** European populations. The *x*-axes indicate the non-reference allele frequencies. The *y*-axes represent the aggregated *R* ^2^ of the imputation results. Vertical dashed lines indicate standard deviations of aggregated *R* ^2^ for each bin. Abbreviation: KBA, Korea Biobank Array; KRG, Korean Reference Genome; ChinaMAP, China Metabolic Analytics Project; GAsP, GenomeAsia 100K Project; TOPMed, Trans-Omics for Precision Medicine.

**FIGURE 4:**
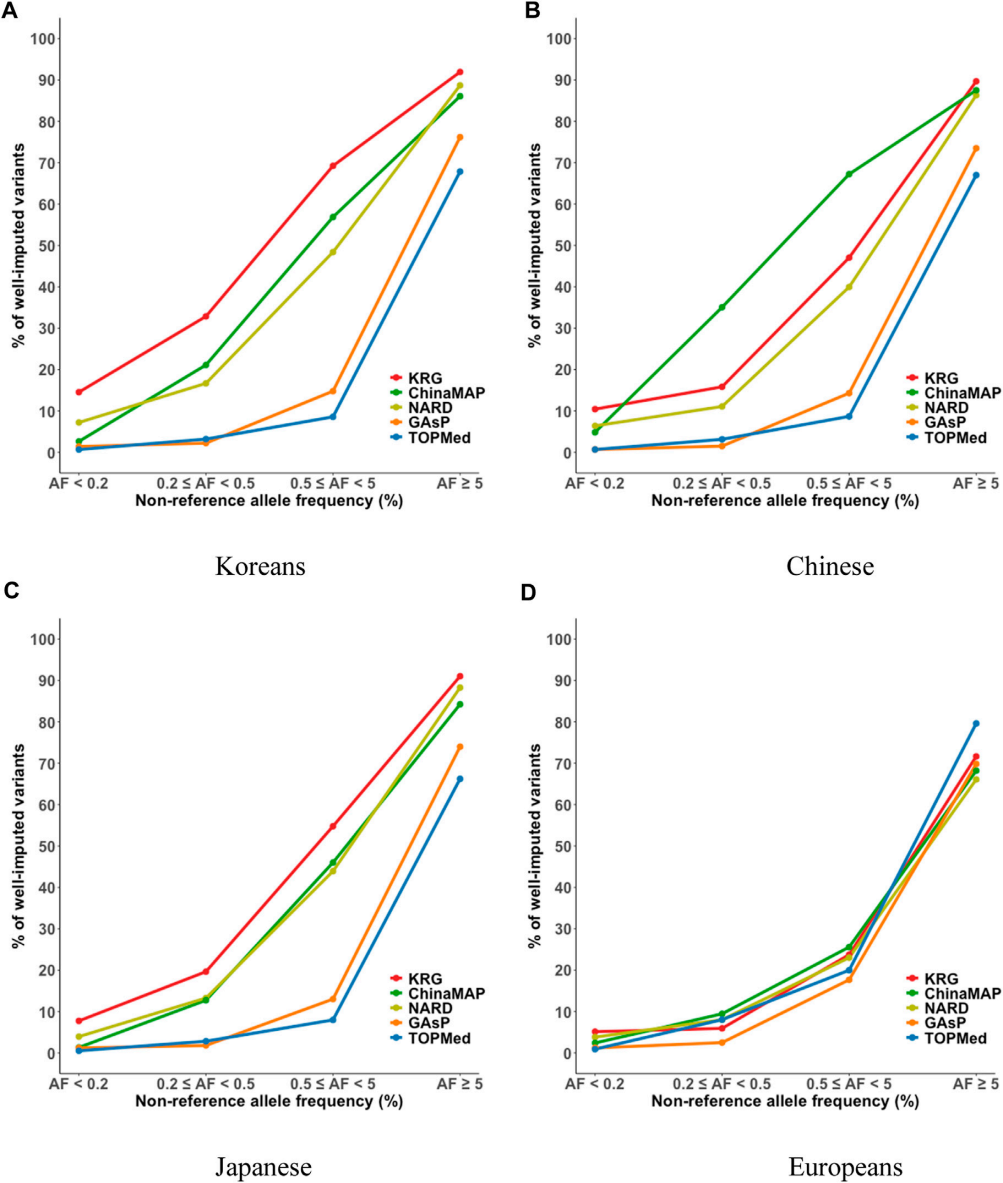
Percent of well-imputed variants, determined by combining reference and genotype panels mimicking the KBA. The % of well-imputed variants (estimated *r* ^2^ ≥ 0.8) was computed for the **(A)** Korean, **(B)** Chinese, **(C)** Japanese, and **(D)** European populations. The *x*-axes indicate the frequencies of non-reference alleles. The *y*-axes represent the % of well-imputed variants among the imputed results. Abbreviations: KBA, Korea Biobank Array; KRG, Korean Reference Genome; ChinaMAP, China Metabolic Analytics Project; GAsP, GenomeAsia 100K Project; TOPMed, Trans-Omics for Precision Medicine.

The imputation performance of the KRG reference panel was evaluated by comparing it with those of pre-existing reference panels, including TOPMed, GAsP, NARD, and ChinaMAP. The characteristics of the reference panels are summarized in Table 2. Eight different types of genotype panels were created based on four different ancestries and the contents of two different SNP microarrays. The two SNP microarrays were the KBA and UKB microarrays, which are optimized for Koreans and Europeans, respectively. The aggregated *R* ^2^ and % of well-imputed variants were used to assess the imputation performances of the reference panels. For KOR genotype panel, the mean aggregated *R* ^2^ results indicated that the KRG panel (*R* ^2^ = 0.78–0.84) and the NARD panel (*R* ^2^ = 0.79–0.83) showed comparable results and better performance over other reference panels (Supplementary Table S2). Although NARD showed increased mean aggregated *R* ^2^ across bins according to allele frequencies, the KRG showed relatively smaller standard deviations of aggregated *R* ^2^ among bins, implying that the variation of imputation quality in the results of KRG would be smaller than other reference panels (Figure 3, Supplementary Figure S3, Supplementary Table S2). Furthermore, the % of well-imputed variants (estimated *r* ^2^ ≥ 0.8) confirmed that the KRG panel was the best performing among the reference panels for Koreans (Figure 4, Supplementary Figure S4, Supplementary Table S3). For genotype panel mimicking KBA, the KRG showed % of well-imputed variants of 91.9% and 69.2% for AF of ≥5% and 0.5–5% for Koreans, respectively. The other reference panels showed % of well-imputed variants of <90% and 60% for AF of ≥5% and 0.5–5% for Koreans, respectively. Unlike reference panels with similar ancestry group such as KRG and ChinaMAP, GAsP showed sharply decreased imputation performance for AF < 5%. This would be due to insufficient number of haplotypes for estimating variants with AF < 5% in Koreans matched among 219 populations of the GAsP panel. Overall, the KRG panel (*n* = 1,490), which comprised samples with a genetically identical background, showed better performance for KOR individuals than the other reference panels that contained up to 65-fold more samples with different genetic backgrounds (TOPMed *n* = 97,256).

**TABLE 2:** Characteristics of reference panels used in this study.

Panel	Mean depth	Number of samples	Number of variants	Ancestry	Imputation server
KRG (pilot phase)	29×	1,490	13,637,761	East Asian (Korean)	-
GAsP	30×	1,654	21,494,814	Asian	https://imputationserver.s*p*h.umich.edu
NARD	24.8x	1,799	27,754,692	Northeast Asian	https://nard.macrogen.com
				
ChinaMAP	40.8×	10,155	59,010,860	East Asian (Chinese)	http://www.mbiobank.com/imputation
TOPMed	30×	97,256	308,107,085	Multi-ethnic	https://imputation.biodatacatalyst.nhlbi.nih.gov

Abbreviations: KRG, Korean References Genome; GAsP, GenomeAsia 100K Project; ChinaMAP, China Metabolic Analytics Project; TOPMed, Trans-Omics for Precision Medicine.

TOPMed showed relatively poor imputation performance for East Asians (KOR, JPT, and CH) compared to those of reference panels with East Asian ancestry including KRG, ChinaMAP, and NARD. These difference in imputation performance may partly be due to variation in LD patterns among populations. We investigated imputation performance in regions with large variations in LD between Korean (KRG) and European (EUR, 1KGP3) populations using varLD software (Ong and Teo, 2010). The varLD analysis revealed several regions with large variation in LD between KRG and EUR such as the 12q24 region, a well known region with population specific genetic architecture for East Asians and Europeans (Kim et al., 2011). We observed differences in imputation quality based on the aggregated *R* ^2^ (R^2^_KRG_ – R^2^_TOPMed_) values among bins of allele frequencies for regions with top 1% variation in LD between populations and remaining 99% of regions. Overall, the top 1% variable region showed that KRG exhibited better imputation accuracy than that of TOPMed (Supplementary Figure S5). This result implies that population specific reference panels enhance association mapping power for regions with population specific genetic architecture such as 12q24.

By comparing multiple reference panels and multi-ethnic genotype panels, our findings further suggested that an optimal imputation performance could be obtained using reference panels with populations genetically close to samples in the genotype panel **(** Figures 3, 4; Supplementary Figures S3, S4) (Kim et al., 2015; Das et al., 2016; GenomeAsia, 2019; Yoo et al., 2019; Jeon et al., 2020; Li et al., 2021). The TOPMed panel showed the best performance for EUR individuals (*R* ^2^ = 0.80–0.87), whereas the ChinaMAP panel performed best for CH individuals (*R* ^2^ = 0.82–0.85) (Supplementary Table S2). For JPT individuals, the NARD (*R* ^2^ = 0.84–0.87) was the most suitable for the Japanese population followed by the ChinaMAP (*R* ^2^ = 0.80–0.84) panel (Supplementary Table S2), although the KRG panel performed the best when considering % of well-imputed variants (91.0% for AF ≥5%, 54.8% for AF 0.5–5%) (Figure 4; Supplementary Figure S4). The imputation performance was also influenced by the contents of SNP microarrays. For example, an optimal imputation performance for the KOR genotype panel was achieved using the KRG reference panel along with the KOR genotype panel that mimicked the KBA (*R* ^2^ = 0.84), an optimized microarray for Koreans, compared with when the genotype panel was used that mimicked the UKB (*R* ^2^ = 0.78) (Figure 3, Supplementary Figure S3; Supplementary Table S2). Similarly, the genotype panels for JPT and CH individuals that mimicked the KBA showed enhanced imputation performance (*R* ^2^ = 0.85) over genotype panels mimicking the UKB array (*R* ^2^ < 0.82) (Supplementary Table 2). However, the EUR genotype panel mimicking the UKB showed better imputation performance (*R* ^2^ = 0.87) than the EUR panel mimicking the KBA (*R* ^2^ = 0.80). These results demonstrated the value of using a population-specific microarray for genome research with a specific population.

### Meta-imputation approach of merging data from the KRG and multi-ethnic reference panels

Although a large-scale population-specific reference panel is an ideal option for maximizing the imputation performance for a specific population, producing tens of thousands of whole-genome sequencing datasets would be a major obstacle, requiring an immense amount of computing time. Alternatively, Yu et al. used a meta-imputation approach to enhance the imputation performance based on imputation results from multiple reference panels, resulting in similar performance with multi-ethnic reference panels with increased sample size (Yu et al., 2022). Meta-imputation would comprehensively increase the imputation performance without assessing individual-level genotype data of the reference panels.

During this study, only the Michigan imputation server (used for the GAsP and 1KGP3 datasets) and TOPMed supported a meta option for an imputation output, which is required for meta-imputation analysis. We performed meta-imputation by merging the imputation results of the KRG, GAsP, TOPMed, and 1KGP3 datasets. The 1KGP3 reference panel was only used to analyze the KOR data because samples from CH, JPT, and EUR subjects were included in 1KGP3.

**FIGURE 5:**
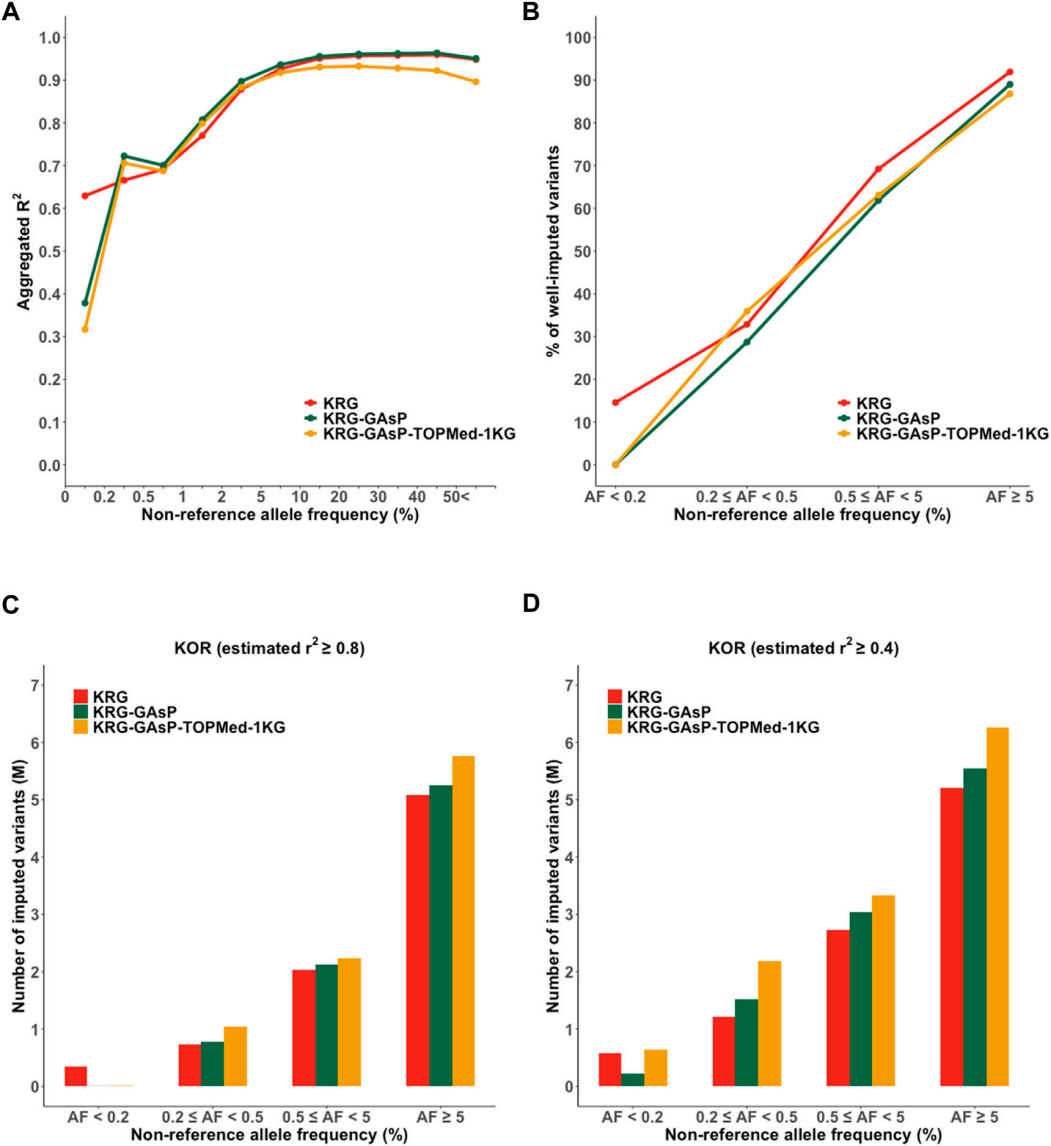
Performance of meta-imputation. Imputation quality metrics for the Korea (KOR) genotype panel mimicking KBA were measured; **(A)** aggregated *R* ^2^, **(B)** % of well-imputed variants (estimated *r* ^2^ ≥ 0.8), and **(C)** number of imputed variants (estimated *r* ^2^ ≥ 0.8), and **(D)** # of imputed variants (estimated *r* ^2^ ≥ 0.4).

The meta-imputation did not enhance imputation performance in overall and showed comparable performance to those of using the single reference panel genetically close to the genotype panel (Figure 5; Supplementary Figure S6, Supplementary Table S4). For example, with the KOR genotype panel mimicking KBA, meta-imputation (KRG- GAsP) and the KRG showed mean aggregated *R* ^2^ as 0.840 and 0.841, respectively. Similarly, with the EUR genotype panel mimicking UKB, meta-imputation (GAsP-TOPMed) and the TOPMed showed mean aggregated *R* ^2^ as 0.870 and 0.873, respectively.

Although the meta-imputation did not greatly increase the overall imputation performance when compared to using the population specific reference panel, however, meta-imputation provided two advantages over using only single reference panel: enhanced imputation accuracy for rare variants and increased number of imputed variants. For the KOR dataset mimicking KBA, meta-imputation showed an increased imputation performance for less common and rare variants (AF = 0.2–5%; *R* ^2^ = 0.72–0.90 for KRG- GAsP) when compared to analyzing the KRG dataset alone (*R* ^2^ = 0.67–0.88) (Supplementary Table S4). Similarly, with the EUR dataset mimicking UKB, meta-imputation (GAsP-TOPMed) showed increased mean aggregated *R* ^2^ (AF = 0.2–5%, *R* ^2^ = 0.80–0.94) when compared to using the TOPMed alone (*R* ^2^ = 0.80–0.91). Furthermore, merging all variants from different reference panels via meta-imputation could expand the spectrum of variants available for analysis. Indeed, meta-imputation increased number of imputed variants. For example, meta-imputation provided an increased number of well-imputed variants (*r* ^2^ ≥ 0.8) by up to 20% (∼9.9 M for KRG-1KG) compared with that available using the KRG dataset alone (8.2 M), especially for variants with AF < 5% (Supplementary Table S4). Of note, the number of well-imputed variants increased by up to 84%, 26%, and 20% for AF bins of 0.2–0.5%, 0.5–5%, and 5%, respectively. Gain of imputed data was further increased when the imputation quality score was relaxed at *r* ^2^ ≥ 0.4, especially for ultra-rare variants with AF ranging from 0.2% to 0.5% (Supplementary Table S4). With the relaxed quality score (*r* ^2^ ≥ 0.4), the KRG provided 1,216,575 imputed variants (55.18% among the variants with AF 0.2–0.5%) yet the meta-imputation (KRG-TOPMed-1KG) provided 2,442,577 imputed variants (81.42%).

By analyzing various combinations of reference panels for meta-imputation, we demonstrated that meta-imputation by merging genetically close reference panels showed an optimal performance as against merging all the available reference panels. For example, with the genotype panel mimicking KBA, genotype panels of East Asian ancestry (KOR and JPT) showed the best performance when the KRG-GAsP was used (*R* ^2^ = 0.84–0.85) (Supplementary Table S4); in contrast, the GAsP-TOPMed combination was found the best for Europeans (*R* ^2^ = 0.79).

Therefore, meta-imputation by using genetically close reference panels can greatly aid in improving the imputation performance and providing additional variants for various forms of analysis.

## 4 Discussion

In this study, we analyzed the whole-genome sequencing data from 1,490 Korean subjects as the pilot phase of the KRG project. The sequencing data from the KRG project showed an enhanced imputation performance over pre-existing imputation reference panels with larger number of samples from different genetic backgrounds than in the KRG dataset. By analyzing various combinations of reference and genotype panels, our findings demonstrated important features for imputation analysis. First, as previously reported, the reference panel that was genetically close to samples in the genotype panel showed the best imputation performance. Second, a population-specific optimized microarray further increased the imputation performance, which enhanced the association-mapping power for a specific population. Finally, the meta-imputation using genetically close reference panels will greatly aid in enhancing the imputation performance with additional variants for subsequent association analyses.

By analyzing various reference panels, we demonstrated that the genetic composition of the reference panel largely affects imputation accuracy. One example is the very poor performance of the KRG when applied to Europeans (Figure 3; Supplementary Figure S3). Although the KRG showed comparable performance with ChinaMAP, NARD, and GAsP when applied to East Asians, the KRG showed more decreased imputation accuracy for Europeans compared to those of ChinaMAP, NARD, and GAsP. The higher imputation accuracy of ChinaMAP, NARD, and GAsP can be the result of an increased genetic diversity that is achieved by including various subpopulations. The ChinaMAP included 8 subpopulations across 27 provinces of China (Cao et al., 2020). NARD and GAsP included 5 and 219 subpopulations, respectively (GenomeAsia, 2019; Yoo et al., 2019). However, GAsP, with the largest number of subpopulations, showed relatively better imputation performance than other East Asian reference panels for Europeans. In contrast, GAsP showed relatively poor imputation accuracy among East Asian reference panels for Koreans. Meanwhile, NARD showed comparable performance to that of the KRG because it had the smallest number of subpopulations (*n* = 5). This implies that increased haplotype diversity, which is the result of a higher number of subpopulations, caused lower imputation accuracy for a specific population due to a relatively smaller number of exactly matched haplotypes. Conversely, increased genetic diversity from numerous subpopulations would provide enhanced imputation accuracy for genetically distant or admixed populations. Collectively, imputation performance can be affected by the genetic compositions of samples in the reference panel. This result will be valuable scientific evidence for imputation analysis in underrepresented and admixed populations.

Imputation analysis is essential for GWAS and *in silico* fine mapping. However, imputation using large scale sequencing data as a reference panel requires an immense amount of computing resources. To facilitate imputation analysis, various imputation servers were introduced including TOPMed, ChinaMAP, NARD, and Michigan imputation servers. Despite these efforts, there are limited dedicated imputation servers for a specific population. For example, there are no publicly available imputation servers for Japanese and Korean populations, major ethnic groups of East Asia. Population specific reference panels and dedicated imputation servers will be valuable resources to the research community, which not only includes those studying specific populations but also those studying underrepresented or admixed populations using meta-imputation approach. In the KRG project, based on the experiences of the pilot project, National Institute of Health, Korea, is planning to open a dedicated imputation server with the interim version of the KRG in 2023.

There are four limitations to our study. First, the KRG pilot phase is still limited in terms of sample size when compared to the pre-existing reference panels. The relatively small number of samples generated thus far in the KRG pilot phase provides a limited number of rare variants for imputation analysis. Over the next couple of years, the KRG will analyze approximately 20,000 whole-genome sequences from Korean subjects. The final phase of the KRG project will provide large volumes of data on rare variants that are potential drug targets and strong genetic modifiers in the Korean population. Second, strict quality control for KRG would lead to limited number of Korean specific variants in the KRG dataset. Therefore, further analysis is warranted to analyze Korea specific variants in aspects of population structure by including more sequenced samples. Third, different levels of sequencing depth in varying projects could cause possible bias in the analysis results. The mapping depth of the KRG was ranged from 8.1x to 50.8x while mean depth of all samples was 29x. Also, the depth was ranged from 24.8x for NARD to 40.8x for ChinaMAP. It is well knwon that higher sequencing depth provides higher accuracy in discovering variants. These varied levels of sequencing depth could affect the accuracy in discovering variants and following imputation analysis. Fourth, we did not use the ChinaMAP and NARD panels for meta-analysis because these did not support the meta-imputation option. As ChinaMAP and NARD showed comparable performance to KRG, meta-imputation of KRG, ChinaMAP, and NARD would be expected to greatly enhance the imputation performance over the meta-imputation approach and reference panels used in this study.

With advances in technologies related to next-generation sequencing, the costs for producing and analyzing whole-genome sequences are expected to decrease sharply within the next few years. A population-specific reference panel would provide an opportunity for exploring variants in the human genome with enhanced imputation accuracy in a cost-effective manner. In addition, various imputation strategies, such as meta-imputation, will further increase the association-mapping power. These efforts will greatly aid in the rapid screening for variants associated with various diseases.

## Data Availability

Publicly available datasets were analyzed in this study. This data can be found here: https://www.koreanchip.org/downloads.

## Abbreviations

1KGP3, 1000 Genomes Project Phase 3; AF, Allele frequency; ChinaMAP, China Metabolic Analytics Project; GAsP, GenomeAsia 100K Project; HRC, Haplotype Reference Consortium; INDEL, Insertions/deletions; JWGS, Japanese whole-genome sequence; KBA, Korea Biobank Array; KGP, Korean Genome Project; KoGES, Korean Genome and Epidemiology Study; KRG, Korean Reference Genome; MAF, Minor-allele frequency; NARD, Northeast Asian Reference Database; SNP, Single-nucleotide polymorphism; TOPMed, Trans-Omics for Precision Medicine; UKB, UK Biobank; VQSR, Variant Quality Score Recalibration.
