# Supplementary material for: Analyzing the Korean reference genome with meta-imputation increased the imputation accuracy and spectrum of rare variants in the Korean population
Source: Front Genet. 2022 Nov 24;13:1008646. doi: 10.3389/fgene.2022.1008646 (PMC9731225; doi:10.3389/fgene.2022.1008646)
Supplement: Supplementary file 1 [file Presentation1.pptx]

## Slide 1
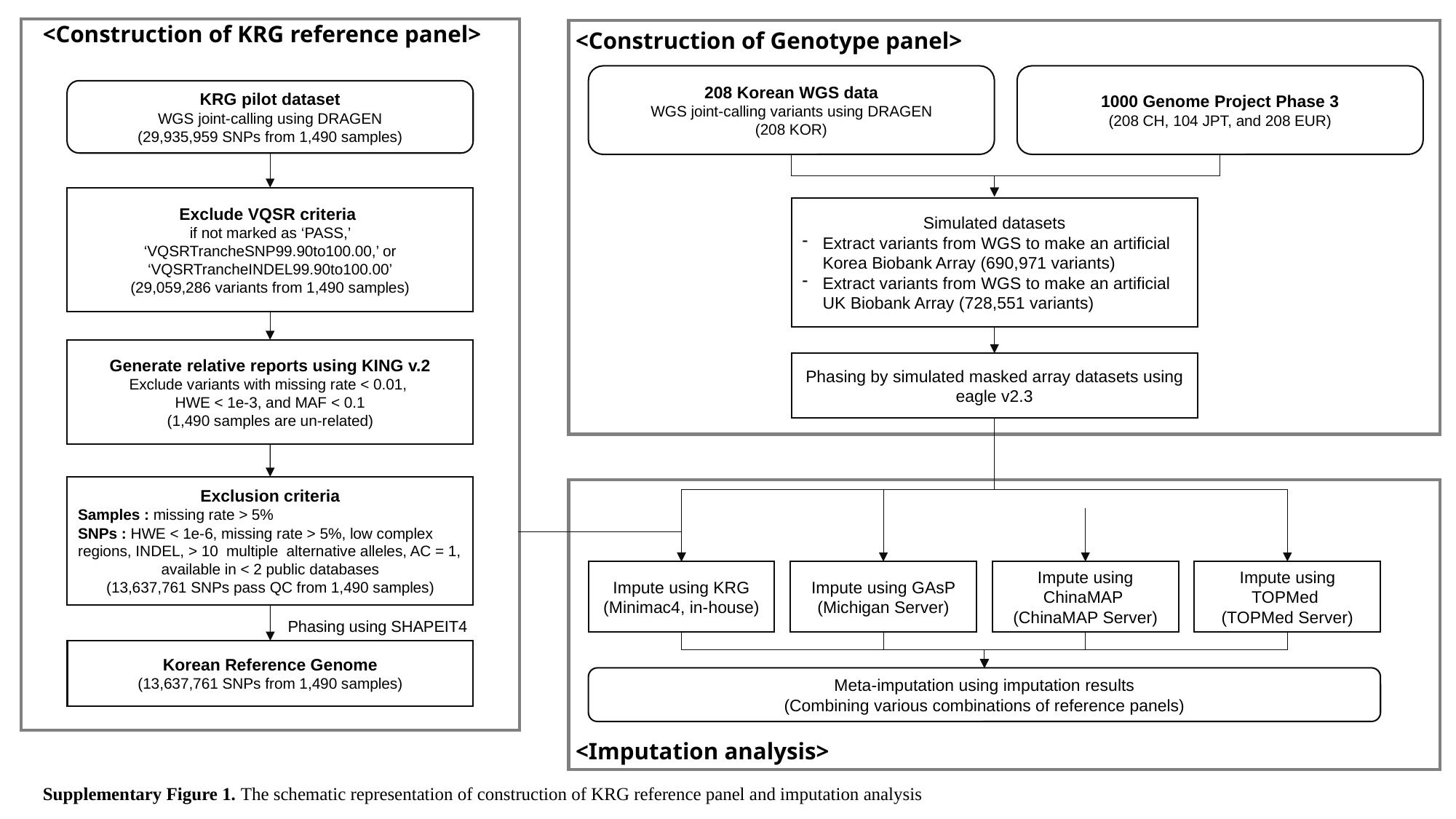

<Construction of KRG reference panel>
<Construction of Genotype panel>
208 Korean WGS data
WGS joint-calling variants using DRAGEN
(208 KOR)
1000 Genome Project Phase 3
(208 CH, 104 JPT, and 208 EUR)
KRG pilot dataset
WGS joint-calling using DRAGEN
(29,935,959 SNPs from 1,490 samples)
Exclude VQSR criteria
if not marked as ‘PASS,’ ‘VQSRTrancheSNP99.90to100.00,’ or ‘VQSRTrancheINDEL99.90to100.00’
(29,059,286 variants from 1,490 samples)
Simulated datasets
Extract variants from WGS to make an artificial Korea Biobank Array (690,971 variants)
Extract variants from WGS to make an artificial UK Biobank Array (728,551 variants)
Generate relative reports using KING v.2
Exclude variants with missing rate < 0.01,
HWE < 1e-3, and MAF < 0.1
(1,490 samples are un-related)
Phasing by simulated masked array datasets using eagle v2.3
Exclusion criteria
Samples : missing rate > 5%
SNPs : HWE < 1e-6, missing rate > 5%, low complex regions, INDEL, > 10 multiple alternative alleles, AC = 1,
available in < 2 public databases
(13,637,761 SNPs pass QC from 1,490 samples)
Impute using ChinaMAP
(ChinaMAP Server)
Impute using KRG
(Minimac4, in-house)
Impute using GAsP
(Michigan Server)
Impute using TOPMed
(TOPMed Server)
Phasing using SHAPEIT4
Korean Reference Genome
(13,637,761 SNPs from 1,490 samples)
Meta-imputation using imputation results
(Combining various combinations of reference panels)
<Imputation analysis>
Supplementary Figure 1. The schematic representation of construction of KRG reference panel and imputation analysis

## Slide 2
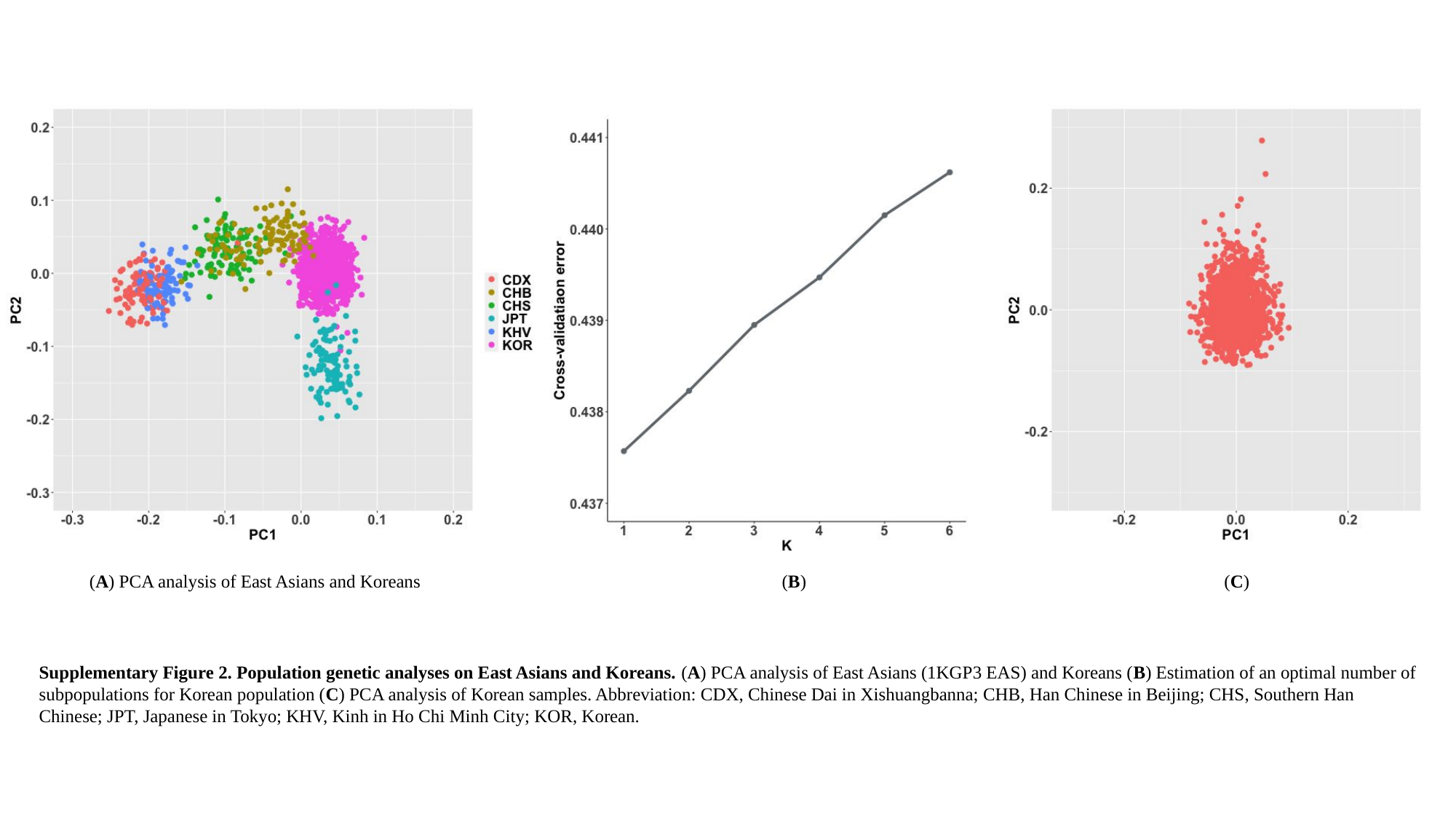

(B)
(C)
(A) PCA analysis of East Asians and Koreans
Supplementary Figure 2. Population genetic analyses on East Asians and Koreans. (A) PCA analysis of East Asians (1KGP3 EAS) and Koreans (B) Estimation of an optimal number of subpopulations for Korean population (C) PCA analysis of Korean samples. Abbreviation: CDX, Chinese Dai in Xishuangbanna; CHB, Han Chinese in Beijing; CHS, Southern Han Chinese; JPT, Japanese in Tokyo; KHV, Kinh in Ho Chi Minh City; KOR, Korean.

## Slide 3
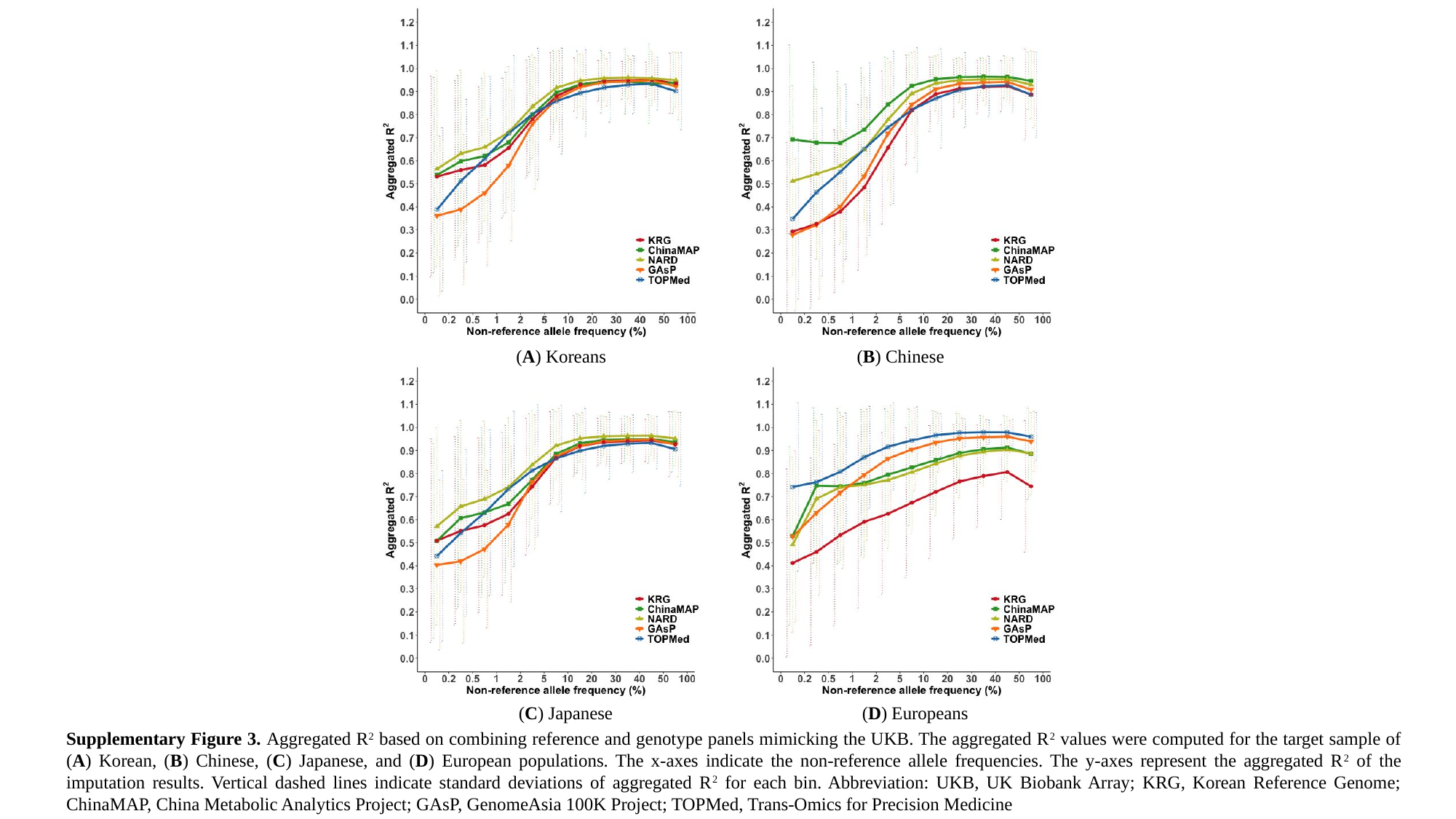

(A) Koreans
(B) Chinese
(C) Japanese
(D) Europeans
Supplementary Figure 3. Aggregated R2 based on combining reference and genotype panels mimicking the UKB. The aggregated R2 values were computed for the target sample of (A) Korean, (B) Chinese, (C) Japanese, and (D) European populations. The x-axes indicate the non-reference allele frequencies. The y-axes represent the aggregated R2 of the imputation results. Vertical dashed lines indicate standard deviations of aggregated R2 for each bin. Abbreviation: UKB, UK Biobank Array; KRG, Korean Reference Genome; ChinaMAP, China Metabolic Analytics Project; GAsP, GenomeAsia 100K Project; TOPMed, Trans-Omics for Precision Medicine

## Slide 4
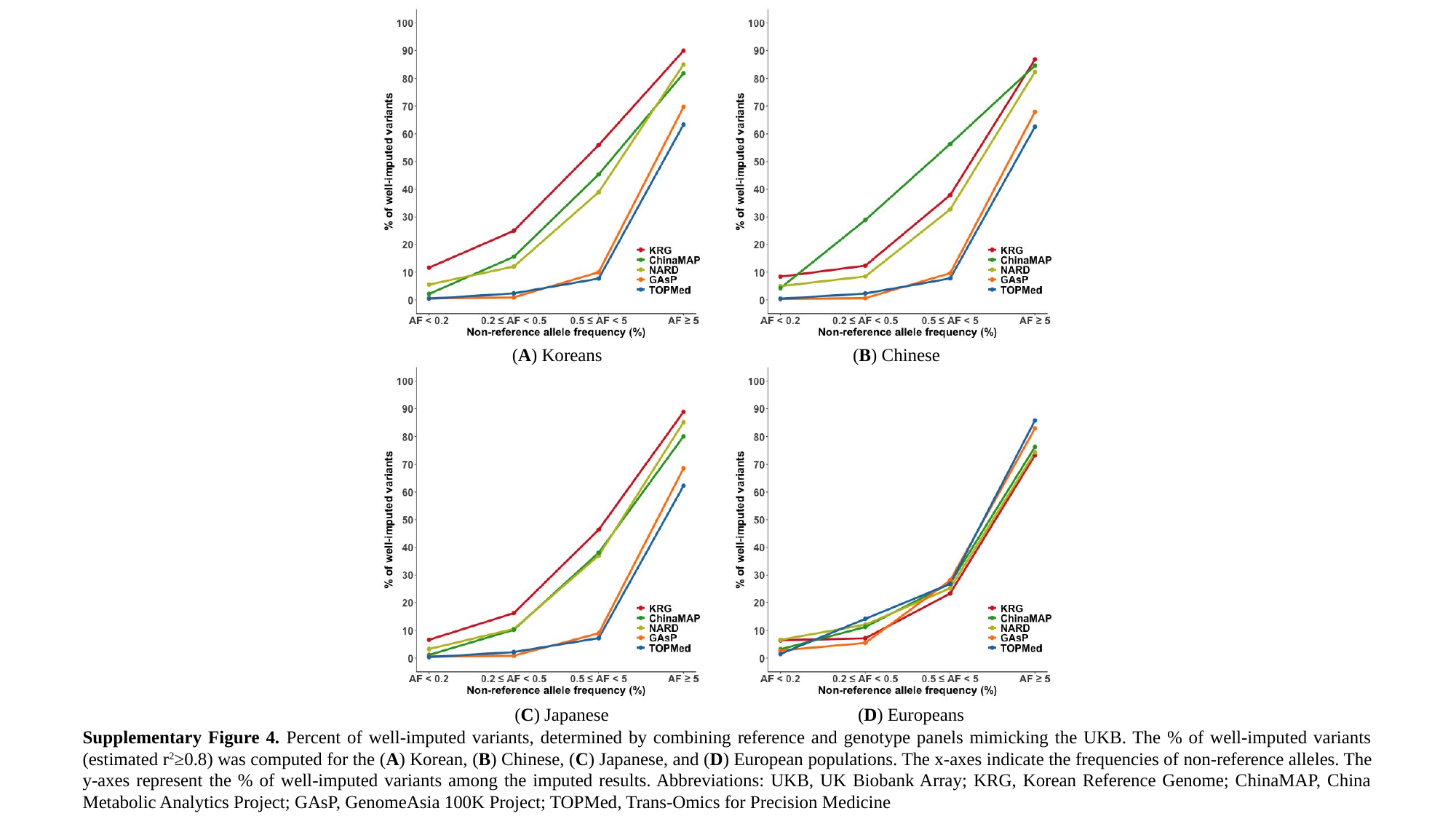

(A) Koreans
(B) Chinese
(C) Japanese
(D) Europeans
Supplementary Figure 4. Percent of well-imputed variants, determined by combining reference and genotype panels mimicking the UKB. The % of well-imputed variants (estimated r2≥0.8) was computed for the (A) Korean, (B) Chinese, (C) Japanese, and (D) European populations. The x-axes indicate the frequencies of non-reference alleles. The y-axes represent the % of well-imputed variants among the imputed results. Abbreviations: UKB, UK Biobank Array; KRG, Korean Reference Genome; ChinaMAP, China Metabolic Analytics Project; GAsP, GenomeAsia 100K Project; TOPMed, Trans-Omics for Precision Medicine

## Slide 5
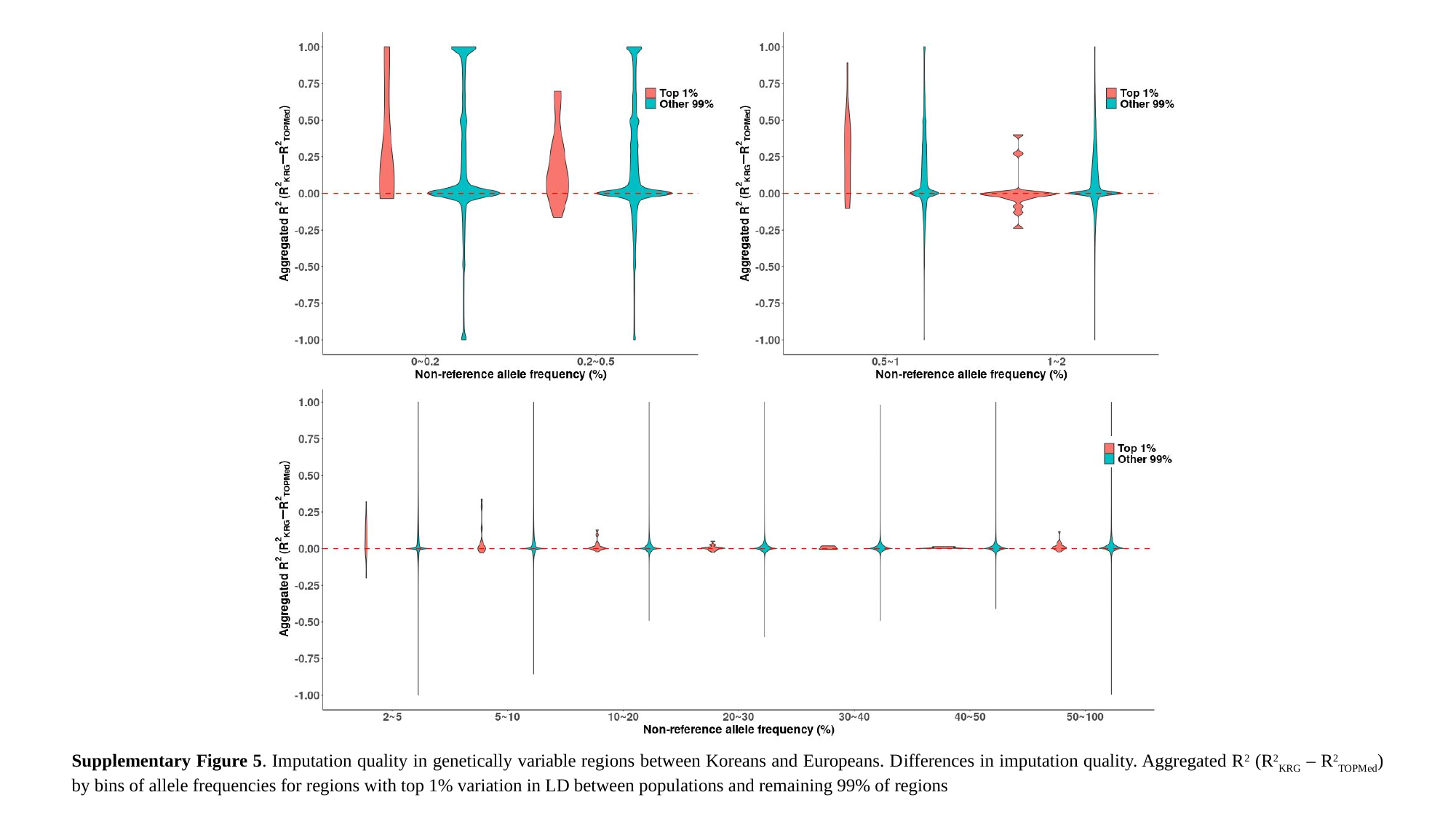

Supplementary Figure 5. Imputation quality in genetically variable regions between Koreans and Europeans. Differences in imputation quality. Aggregated R2 (R2KRG – R2TOPMed) by bins of allele frequencies for regions with top 1% variation in LD between populations and remaining 99% of regions

## Slide 6
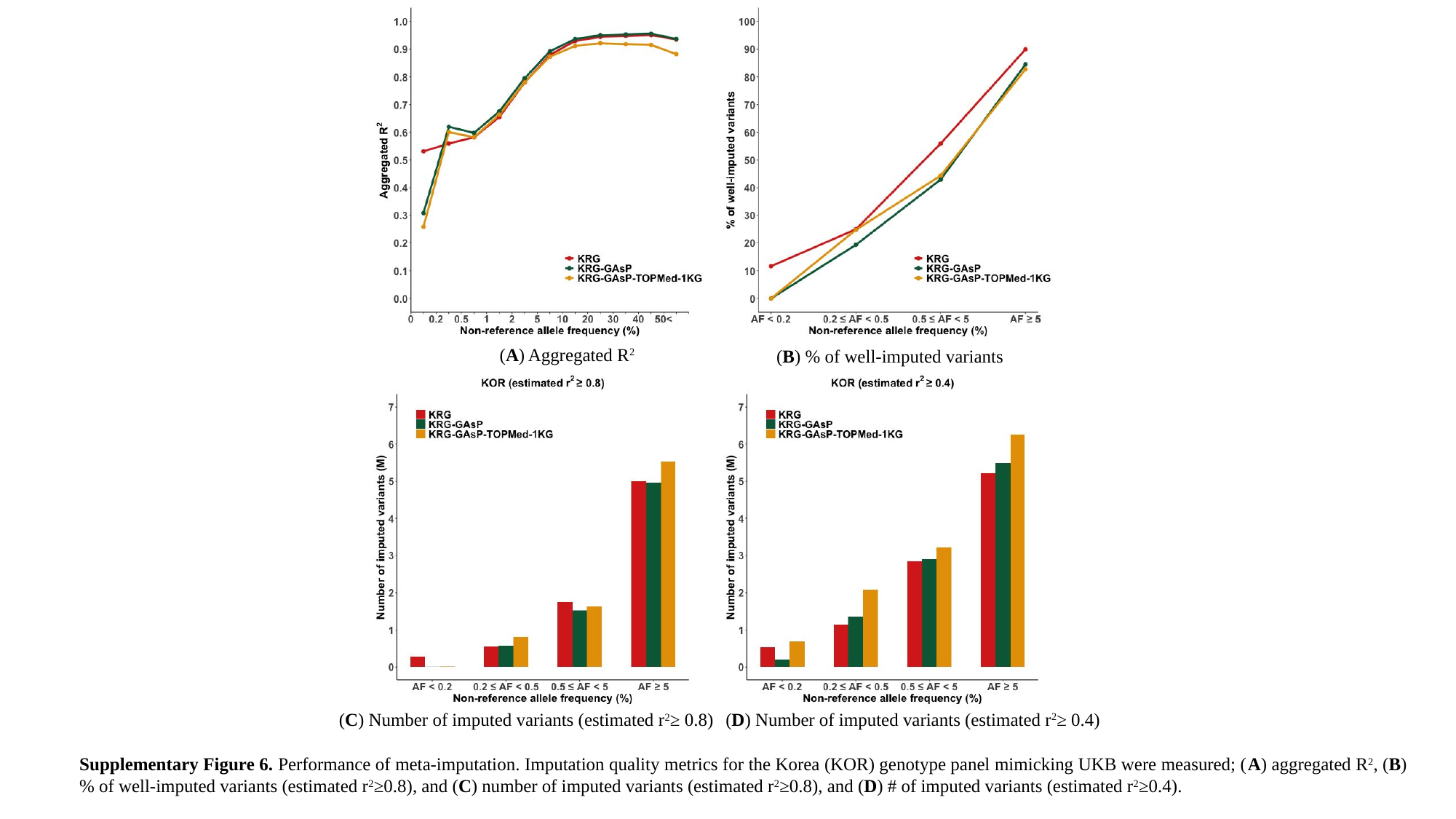

(A) Aggregated R2
(B) % of well-imputed variants
(D) Number of imputed variants (estimated r2≥ 0.4)
(C) Number of imputed variants (estimated r2≥ 0.8)
Supplementary Figure 6. Performance of meta-imputation. Imputation quality metrics for the Korea (KOR) genotype panel mimicking UKB were measured; (A) aggregated R2, (B) % of well-imputed variants (estimated r2≥0.8), and (C) number of imputed variants (estimated r2≥0.8), and (D) # of imputed variants (estimated r2≥0.4).
